# CDK4/6 inhibitors display a class effect in inducing differentiation of neuroblastoma cells

**DOI:** 10.12688/wellcomeopenres.23190.2

**Published:** 2025-09-03

**Authors:** Kirsty M. Ferguson, Fiona M. Y. Abou Grealy, Anna Philpott

**Affiliations:** 1 Jeffrey Cheah Biomedical Centre, Cambridge Stem Cell Institute, Cambridge, CB2 0AW, UK; 2 University of Cambridge Department of Oncology, Cambridge, CB2 0XZ, UK

**Keywords:** neuroblastoma, CDK4/6, retinoic acid, differentiation, palbociclib, abemaciclib, ribociclib

## Abstract

**Background:**

Neuroblastoma is the most common extracranial solid tumour in infants and children, accounting for approximately 15% of paediatric cancer mortality. These tumours are unique in that a subset, namely stage MS, frequently undergo spontaneous regression or differentiation. Differentiation therapy, where cancer cells are re-routed back down their correct developmental pathway, is therefore a promising therapeutic avenue. We have previously shown that the CDK4/6 inhibitor palbociclib induces both decreased proliferation and enhanced neuronal differentiation of neuroblastoma cells *in vitro*. When combined with retinoic acid, already used clinically for maintenance therapy, this differentiation is enhanced.

**Methods:**

Here, we investigate two additional CDK4/6 inhibitors, abemaciclib and ribociclib, to induce differentiation of the relapsed, high-risk MYCN-amplified neuroblastoma cell line SK-N-BE(2)C, with and without retinoic acid. We culture SK-N-BE(2)C cells in both adherent and three-dimensional culture and monitor proliferation and differentiation using readouts including live-imaging, immunocytochemistry, qRT-PCR and EdU incorporation.

**Results:**

We find the CDK4/6 inhibitors palbociclib, abemaciclib and ribociclib all enhance retinoic acid-induced differentiation in both adherent SK-N-BE(2)C cells and 3D spheroids.

**Conclusions:**

CDK4/6 inhibitors display a class effect in inducing neuronal differentiation together with retinoic acid, both in adherent neuroblastoma cell lines and three-dimensional tumour spheroids. This is an important consideration for potentially developing CDK inhibitor-induced differentiation as a therapy in the clinic.

## Plain language summary

Neuroblastoma is the most common solid tumour outside of the brain in children and infants. These tumours happen when developing cells in the sympathetic nervous system fail to specialise properly and begin dividing uncontrollably. Rerouting neuroblastoma cells back down their correct pathway is a promising therapeutic strategy that may present fewer long-lasting side-effects than therapies that directly kill cells.

We have previously found that a drug called palbociclib, which interferes with cell division and is used to treat some types of breast cancer, triggers neuroblastoma cells to specialise into neurons. Combining this with retinoic acid, a derivative of vitamin A already used to prevent neuroblastoma tumours regrowing, enhances this effect even more.

In this research article, we wanted to see if this effect is specific to the drug palbociclib. To do this, we test two other drugs which interfere with cell division in the same way as palbociclib. We find that all three drugs, together with retinoic acid, trigger aggressive neuroblastoma cells to specialise into neurons. These drugs therefore have a ‘class effect’. This is important knowledge for developing this therapy from the bench to the clinic, where the drug used must be balanced with factors such as efficacy, availability and cost.

## Introduction

Neuroblastoma is the most common extracranial solid tumour in infants and children, accounting for approximately 15% of paediatric cancer mortality. Novel, kinder therapies are desperately needed to treat affected children while minimising long-term side-effects. Neuroblastoma is a disease of development gone awry, where developing sympathoadrenal cells go down the wrong path. A subset of tumours, namely International Neuroblastoma Risk Group (INRG) stage MS, frequently undergo spontaneous regression into benign ganglioneuroma ^1^. Re-routing neuroblastoma cells back down their correct developmental path is therefore a promising therapeutic strategy. Previously we found that the CDK4/6 inhibitor, palbociclib (PB, Ibrance, Pfizer; PD-0332991) both decreases proliferation and induces neuronal differentiation of adrenergic (ADRN) neuroblastoma cells. When combined with retinoic acid (RA), already used clinically in high-risk neuroblastoma as maintenance therapy for minimal residual disease, the oncogenic core regulatory circuitry was reset, proliferation was further reduced, and differentiation further enhanced, compared to PB or RA treatment alone ^2^.

Dysregulation of the Cyclin D-CDK4/6-INK4-Rb pathway and subsequent unchecked cell proliferation, is a common feature of human cancers; CDK4/6 activity is therefore a key target to attenuate tumour growth. Palbociclib (PD-0332991, Pfizer) was the first CDK4/6-specific inhibitor to be discovered and to show clinical efficacy ^3– 7^. Ribociclib (LEE011, Novatis and Astex Pharmaceuticals) and abemaciclib (LY2835219, Eli Lilly and Company) later followed. All three are now FDA-approved for combinatorial treatment of HR-positive and HER2-negative breast cancers with endocrine therapy ^8^, each displaying unique *in vitro* specificity, pharmacokinetics and clinical toxicity profiles ^3^. For example, while abemaciclib is structurally different from palbociclib and ribociclib, abemaciclib and ribociclib have shown a higher potency to CDK4 than CDK6, while palbociclib shows no difference in potency between the two CDKs ^9^. While all drugs are orally available, palbociclib and ribociclib are dosed twice daily, with a dose interruption due to grade 3–4 neutropenia observed in 60% of patients ^10, 11^; in contrast, abemaciclib is dosed twice daily, continuously, and while only 21% of patients experience grade 3–4 neutropenia, 10% develop a different side-effect of grade 3 diarrhoea ^3, 10, 11^.

Several clinical trials are now ongoing investigating CDK4/6 inhibitors in paediatric cancers. For example, a phase 1/2 study is underway to evaluate palbociclib in combination with irinotecan and temozolomide or in combination with topotecan and cyclophosphamide in paediatric patients with recurrent or refractory solid tumours (Clinical trials GovID: NCT03709680). The efficacy and safety of ribociclib in combination with topotean and temozolamide (TOTEM) in paediatric patients with relapsed or refractory neuroblastoma and other solid tumours is also being investigated (Clinical trials GovID: NCT05429502), as is abemaciclib in combination with other treatments in children with solid tumours such as neuroblastoma (ClinicalTrials.gov ID NCT04238819). In this study, we set out to investigate the ability of these three CDK4/6 inhibitors: palbociclib (PB), abemaciclib (ABE) and ribociclib (RIBO), to induce differentiation of the relapsed, high-risk MYCN-amplified neuroblastoma cell line SK-N-BE(2)C, with and without retinoic acid (RA). We find that CDK4/6 inhibitors display a class effect in inducing neuronal differentiation together with retinoic acid, both in two-dimensional and three-dimensional *in vitro* neuroblastoma cell cultures. 

## Methods

### Cell line maintenance and drug treatment

Neuroblastoma cell lines SK-N-BE(2)C and SH-EP were cultured in DMEM-F12 with L-glutamine (Sigma, D8437) with 10% FBS (PAN-Biotech, P40-37500) and 1% Penicillin-Streptomycin (Sigma, P0781), with media refreshed every 2–3 days. At least every 3 months cells were confirmed to be *Mycoplasma* negative. Cells were seeded as in 2 to induce aggregation into spheroids. On reaching 200–400 μm in diameter ^12^, drug treatment of spheroids was commenced. All drugs were dissolved in DMSO (Santa Cruz, sc-358801). Palbociclib (PD-0332991 HCl, SelleckChem, S1116) was used at 1 μM. All-trans retinoic acid (Sigma, R2625) was used at 10 μM. Ribociclib (SelleckChem, S7440) was used at 2 μM. Abemaciclib (SelleckChem S5716) was used at 0.2 μM.

### Immunocytochemistry

Cells were fixed with 4% paraformaldehyde (PFA, ThermoScientific, 15424389) for 10 min at room temperature (RT) then incubated in PBST (PBS with Triton X-100 0.2% (Sigma, X100)) for 10 min at RT. Following incubation in blocking solution (PBS-BSA 3% (Fisher BioReagents, BP9706-100) 0.2% Triton X-100) for 1 hr at RT, cells were incubated overnight with primary antibody at 4 °C in blocking solution (TUBB3, Biolegend 801202, 1:1000). Following washes in PBST, cells were incubated with secondary antibody (Thermo Fisher Scientific, A11029) in blocking solution (1 hr, RT) and washed again. Nuclear counterstaining was performed using DAPI (Abcam, ab228549) (1:10000 in PBST, 15 min, RT). Imaging was performed on a Leica DMI 6000B Matrix microscope with Leica LAS X software ( https://www.leica-microsystems.com/products/microscope-software/p/leica-las-x-ls/). Images were processed using FIJI, an open-source platform for biological image analysis (version: 2.14.0/1.544f, https://imagej.net/software/fiji/downloads). 3D spheroid cultures were stained using the same protocol. Spheroids were mounted with ProLong Gold antifade reagent (ThermoFisher, P36930) onto glass slides and imaged on Andor Revolution Nikon spinning disk confocal. Spheroid images were processed in FIJI as maximum intensity projections of Z stacks.

### Western blotting

Protein lysates were prepared in RIPA buffer (Sigma, R0278) on ice for 20 min, followed by centrifugation (13,000 rpm, 10 min). Following quantification using the BCA method (ThermoScientific, A55864), 15μg protein was separated on a 4–12% BisTris gel (Invitrogen, NP0301) and transferred to a nitrocellulose membrane (Bio-Rad, 1620115). Membranes were blocked with 5% milk-TBS-T (Serva, 42590) then incubated with primary antibodies diluted at 1:1000 in blocking solution (RB, CST 9309S, pRB, CST 9308S, TBP, Proteintech 22006-1-AP) at 4°C overnight, followed by washing in TBST and incubation with HRP-linked secondary antibodies at 1:10000 in blocking solution (Sigma, NA931 and NA934) for 1 hr at RT. Protein bands were visualised on X-ray films after incubation with ECL chemiluminescent substrate (Thermo Fisher Scientific, 32132). Films were scanned and further processed using FIJI.

### EdU incorporation assay

On the 4 ^th^ day of the 5-day drug treatment, media supplemented with 10 μM EdU was added to the cells for 24 hr. Fixation was performed in 4% PFA (10 min, RT). EdU staining was performed with the Click-iT EdU assay kit (Life Technologies, 15224959), followed by DAPI nuclear counterstaining. After tiled imaging using the Leica DMI 6000B Matrix microscope (~44 fields of view), FIJI image thresholding was used to create binary images and FIJI particle analysis function was used to determine the total cell number from DAPI staining and quantify % EdU positive cells. As standard for fluorescence imaging analysis, the same imaging settings were used for all treatment groups and technical replicates to enable internal comparison.

### Confluence analyses and live-imaging

Confluence analysis and live-imaging were performed using the Incucyte® Live-Cell Analysis System (Sartorius). For each treatment, technical triplicates on a 24-well plate were imaged every 6 hours for 5 days. All live imaging was performed three times independently. Confluency was also visualised by crystal violet staining. Cells were fixed in 6 well plates on day 5 with 4% PFA (10 min, RT), stained with 0.5% aqueous crystal violet staining solution (Sigma, V5265 - 30 min, RT) and washed with deionised water.

### Quantitative RT-PCR (qRT-PCR)

The RNeasy Mini kit (Qiagen, 74104) and QuantiTect Reverse Transcriptase Kit (Qiagen, 205311) were used for RNA extraction and reverse transcription, respectively. qRT-PCR was performed on an Applied Biosystems StepOne™ Real-Time PCR system using gene-specific primers (see Table S1, Extended data) and SYBR™ Green Master Mix (Applied Biosystems, A25742). Technical replicates were run to ensure pipetting accuracy and data analysed using the ddCt method, normalised to the housekeeping gene, TBP. Data are shown as mean +/- 95% CI Fold change, where fold change = 1 for the calibrator. Before data transformation, statistics were performed and error bars calculated from ddCt values.

### Statistical analysis

Statistical analyses were made on GraphPad Prism (version 10.2.2 for macOS, https://www.graphpad.com) from at least three independent experiments unless noted (n numbers in figure legends). R is a free software alternative. GraphPad Prism Viewer is free and allows visualisation of data, analyses and graphs. Different passages of SK-N-BE(2)C plated in independent experiments were taken as biological replicates.

## Results

### The CDK4/6 inhibitors palbociclib, abemaciclib and ribociclib all reduce proliferation and induce differentiation of adherent SK-N-BE(2)C neuroblastoma cells

**Figure 1.**
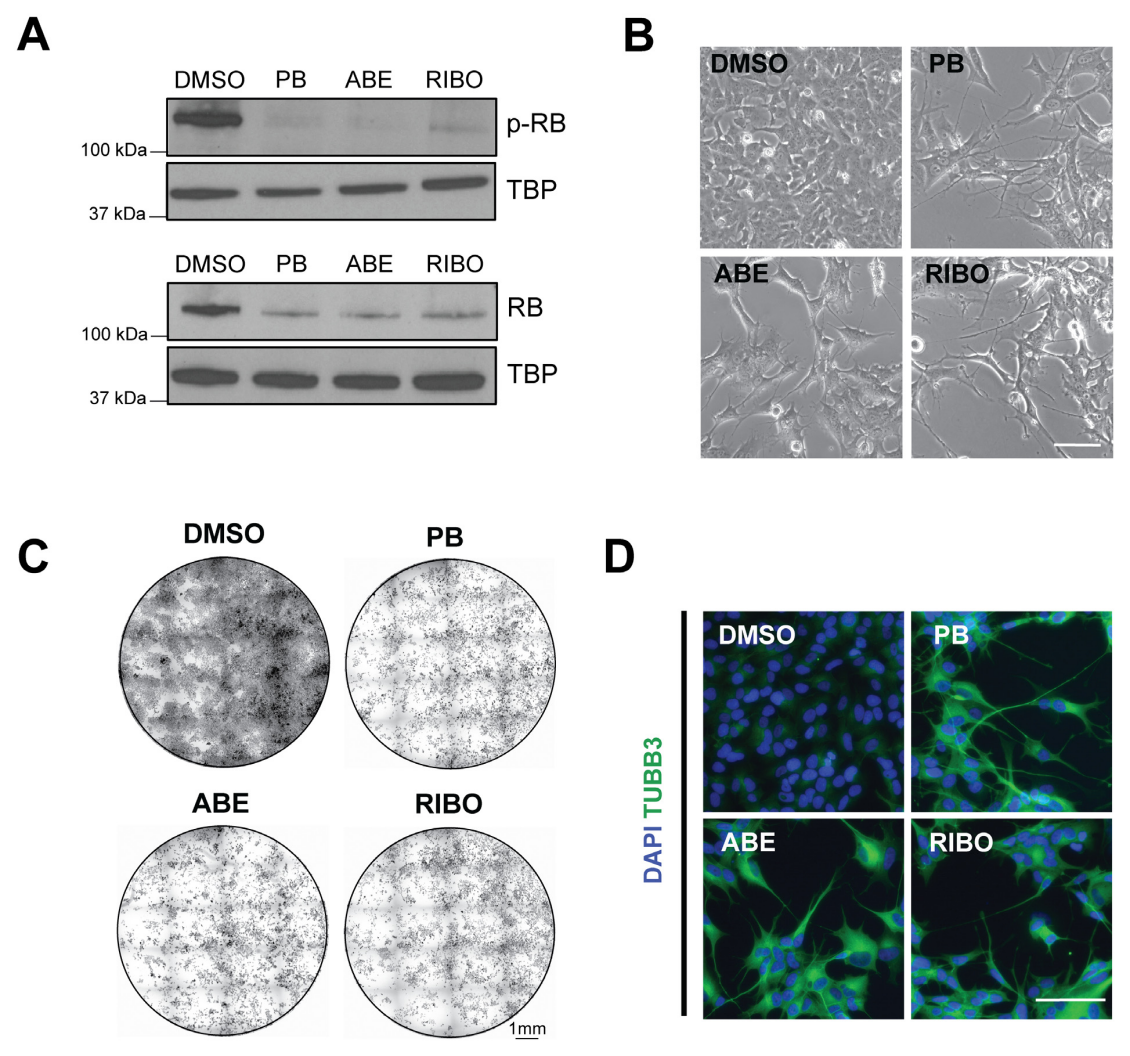
The CDK4/6 inhibitors palbociclib, abemaciclib and ribociclib all reduce proliferation and induce differentiation of adherent SK-N-BE(2)C neuroblastoma cells. ( **A**) Western blot analysis of phospho-RB and total RB protein levels in SK-N-BE(2)C cells treated with palbociclib (PB, 1 μM), abemaciclib (ABE, 0.2 μM), ribociclib (RIBO, 2 μM) or DMSO vehicle control for 24 h. TBP = housekeeping loading control. The same concentrations are used throughout the manuscript. ( **B**) Representative phase-contrast images of SK-N-BE(2)C cells treated with palbociclib (PB), abemaciclib (ABE), ribociclib (RIBO) or DMSO vehicle control for 5 days. Representative of n = 3 biological replicates. Scale bar: 100 μm. ( **C**) Crystal violet staining of SK-N-BE(2)C cells treated with palbociclib (PB), abemaciclib (ABE), ribociclib (RIBO) or DMSO vehicle control for 5 days. Representative of n = 3 biological replicates. Scale bar: 1 mm. ( **D**) Immunocytochemistry analysis of neuronal marker βIII-tubulin (TUBB3, green) expression in SK-N-BE(2)C cells following 5 days of palbociclib (PB) , abemaciclib (ABE), ribociclib (RIBO) or DMSO vehicle control treatment. Scale bar: 100 μm. DAPI nuclear counterstain (blue). Representative of n = 3 biological replicates. Note: Figure 1 data forms part of dataset shown in Figure 2.

We first set out to compare the effects of the three FDA-approved CDK4/6 inhibitors palbociclib (PB), abemaciclib (ABE) and ribociclib (RIBO) on the ADRN-type neuroblastoma cells SK-N-BE(2)C. SK-N-BE(2)C cells are derived from a relapsed tumour that is MYCN-amplified and therefore representative of high-risk disease. Previously, a dosage of 1 μM palbociclib was used, a standard dosage used in cellular studies that is similar to the IC50 ^2^. We therefore ascertained the IC50 of ribociclib and abemaciclib in SK-N-BE(2)C cells (2 μM and 0.2 μM, respectively) for comparison to palbociclib at 1 μM (Figure S1A, Extended data). An indicator of successful CDK4/6 inhibition is reduced phosphorylation of RB, a tumour suppressor that blocks the G1-S transition ^13^. We therefore first confirmed that SK-N-BE(2)C cells treated with PB, ABE or RIBO for 24h resulted in RB hypo-phosphorylation ( Figure 1A). A reduction in total RB was also observed, as reported in neuroblastoma cells upon CDK4/6 knock-down ^14^.

After 5 days of treatments with each CDK4/6 inhibitor, we observed a reduction in proliferation compared to the DMSO control, seen by imaging and crystal violet analysis ( Figure 1B,C). Previously, we observed neuronal differentiation co-incident with reduced proliferation upon PB treatment, visible as neurite outgrowth ^2^. We also observed this phenomenon upon treatment of SK-N-BE(2)C cells with each of the three CDK4/6 inhibitors. Live imaging showed a change of cell morphology during the 5-day treatment, accompanied by negligible cell death (Movie S1, Extended data). The resulting morphology was also visible by immunocytochemistry for the classical neuronal marker βIII-tubulin (TUBB3), whose expression increased upon CDK4/6 inhibition ( Figure 1D). By contrast, MES-type neuroblastoma cells, SH-EP, did not show signs of neuronal differentiation with any CDK4/6 inhibitor treatment, in agreement with our previous study (Figure S1B,C, Extended data). Together these data show that the three CDK4/6 inhibitors palbociclib, abemaciclib and ribociclib are capable of reducing proliferation and inducing differentiation of the neuroblastoma cell line SK-N-BE(2)C *in vitro*, without extensive cell death.

### The CDK4/6 inhibitors palbociclib, abemaciclib and ribociclib all enhance retinoic acid-induced differentiation in adherent SK-N-BE(2)C cells

**Figure 2.**
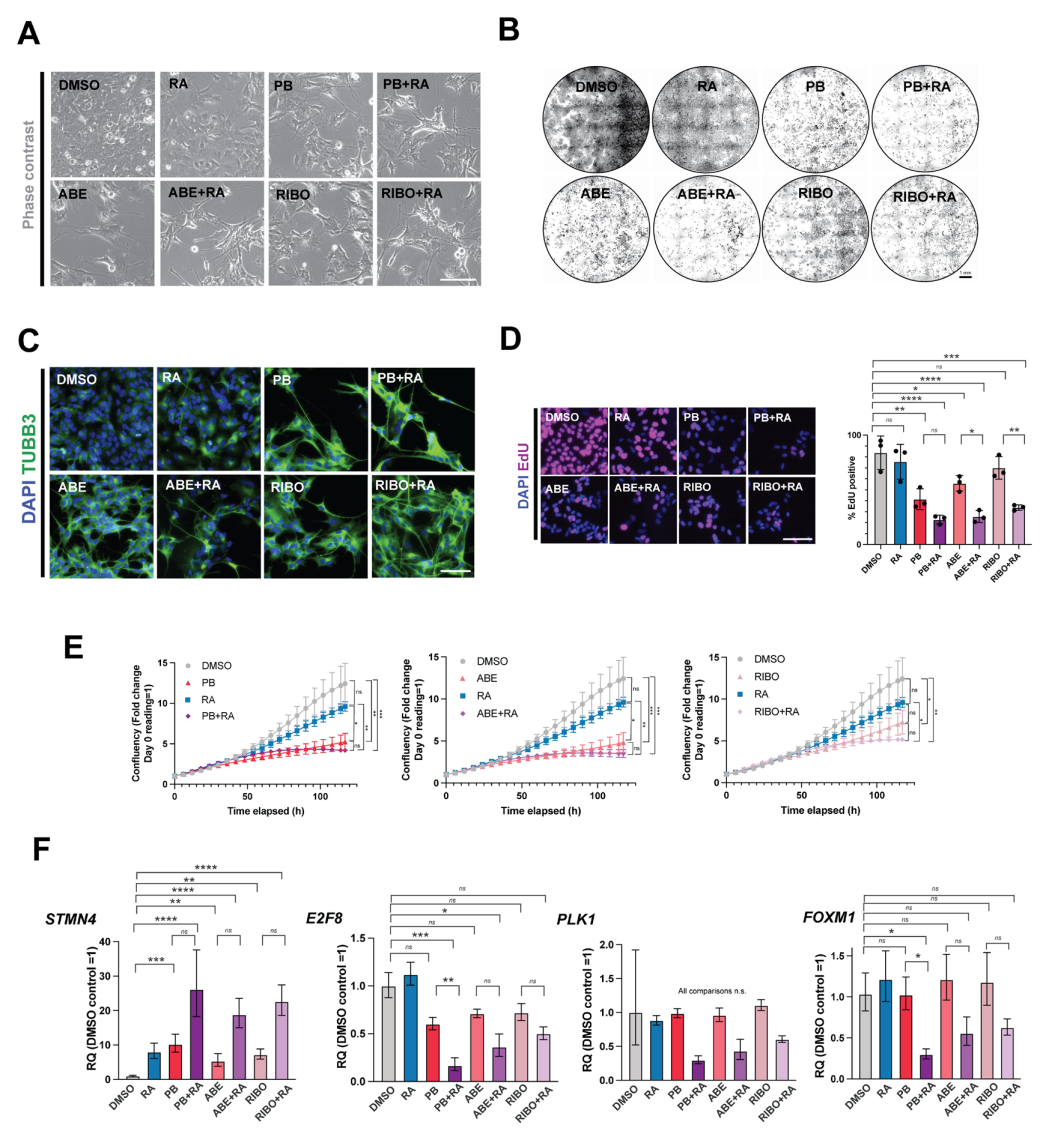
The CDK4/6 inhibitors palbociclib, abemaciclib and ribociclib all enhance retinoic acid-induced differentiation in adherent SK-N-BE(2)C cells. ( **A**) Representative phase-contrast images of SK-N-BE(2)C cells treated with palbociclib (PB, 1 μM), abemaciclib (ABE, 0.2 μM), ribociclib (RIBO, 2 μM), retinoic acid (RA, 10 μM) or a combination of PB/ABE/RIBO + RA, or DMSO vehicle control for 5 days. Representative of n = 3 biological replicates. Scale bar: 50 μm. The same concentrations are used throughout the manuscript. ( **B**) Crystal violet staining of SK-N-BE(2)C cells treated with palbociclib (PB), abemaciclib (ABE), ribociclib (RIBO), retinoic acid (RA) or a combination of PB/ABE/RIBO + RA, or DMSO vehicle control for 5 days. Representative of n = 3 biological replicates. Scale bar: 1 mm. ( **C**) Immunocytochemistry analysis of neuronal marker βIII-tubulin (TUBB3, green) expression in SK-N-BE(2)C cells treated with palbociclib (PB), abemaciclib (ABE), ribociclib (RIBO), retinoic acid (RA) or a combination of PB/ABE/RIBO + RA, or DMSO vehicle control for 5 days. Scale bar: 100 μm. DAPI nuclear counterstain (blue). Representative of n = 3 biological replicates. ( **D**) Left: representative fluorescent images of EdU incorporation following a 24-h pulse in untreated SK-N-BE(2)C cells and cells treated with palbociclib (PB), abemaciclib (ABE), ribociclib (RIBO), retinoic acid (RA) or a combination of PB/ABE/RIBO + RA, or DMSO vehicle control for 5 days. Scale bar: 100 μm. Right: analysis of % EdU-positive cells. Mean ± SD. n = 3 biological replicates, each in technical triplicate. ^*^p ≤ 0.05; ^**^p ≤ 0.01, ^***^p ≤ 0.001 one-way ANOVA with Tukey’s multiple comparison test. Selected comparisons shown for ease of visualisation. ( **E**) Confluence analysis SK-N-BE(2)C cells treated with palbociclib (PB), abemaciclib (ABE), ribociclib (RIBO), retinoic acid (RA) or a combination of PB/ABE/RIBO + RA, or DMSO vehicle control for 5 days. Mean ± SD, n=3 biological replicates (each with n=3 technical replicates). Confluency presented as fold change of day 0 reading, where day 0 reading = 1). ^*^p ≤ 0.05; ^**^p ≤ 0.01, ^***^p ≤ 0.001 one-way ANOVA with Tukey’s multiple comparison test at Day 5 timepoint. ( **F**) qRT-PCR analysis of *STMN4, E2F8, PLK1* and *FOXM1* expression levels in SK-N-BE(2)C cells treated with palbociclib (PB), abemaciclib (ABE), ribociclib (RIBO), retinoic acid (RA) or a combination of PB/ABE/RIBO + RA, or DMSO vehicle control for 5 days. n = 3 biological replicates. Mean ± 95% CI. ^*^p ≤ 0.05; ^**^p ≤ 0.01, ^***^p ≤ 0.001; and ^****^p ≤ 0.0001, one-way ANOVA with Tukey’s multiple comparison test. Selected comparisons shown for ease of visualisation.

Retinoic acid (RA) is a differentiation agent already used as standard of care in maintenance therapy for high-risk neuroblastoma. RA has been found to epigenetically reset the core regulatory circuit of ADRN-type neuroblastoma cells ^15^ and is viewed as a positive standard for differentiation induction. We found that dual treatment with retinoic acid and palbociclib further suppressed proliferation and enhanced differentiation compared to either drug alone ^2^. We therefore next sought to determine if retinoic acid enhances differentiation in combination with palbociclib alone, or with other CDK4/6 inhibitors. For each of the three CDK4/6 inhibitors, CDK4/6 inhibition or dual treatment with retinoic acid showed a greater decrease in proliferation compared to DMSO or RA alone as shown by crystal violet staining, EdU analysis and live-imaging confluency analyses ( Figure 2 A,B,D,E, Movie S2, Extended data). The decrease in proliferation by each CDK4/6 inhibitor was consistently enhanced by combinatorial treatment with retinoic acid.

Upon treatment with either CDK4/6 inhibitor alone, or in combination with RA, ICC showed an increase in TUBB3 expression and neurite extension (compared to DMSO or RA treatment) ( Figure 2C). qRT-PCR analysis revealed a greater increase in expression of the differentiation marker *STMN4*, and a greater decrease in expression of the proliferative markers *E2F8, PLK1* and *FOXM1* ^16, 17^, upon treatment of cells with each CDK4/6 inhibitor plus RA, compared to treatment with each CDK4/6 inhibitor alone ( Figure 2F).

### The CDK4/6 inhibitors palbociclib, abemaciclib and ribociclib all enhance retinoic acid-induced differentiation in 3D SK-N-BE(2)C spheroids

**Figure 3.**
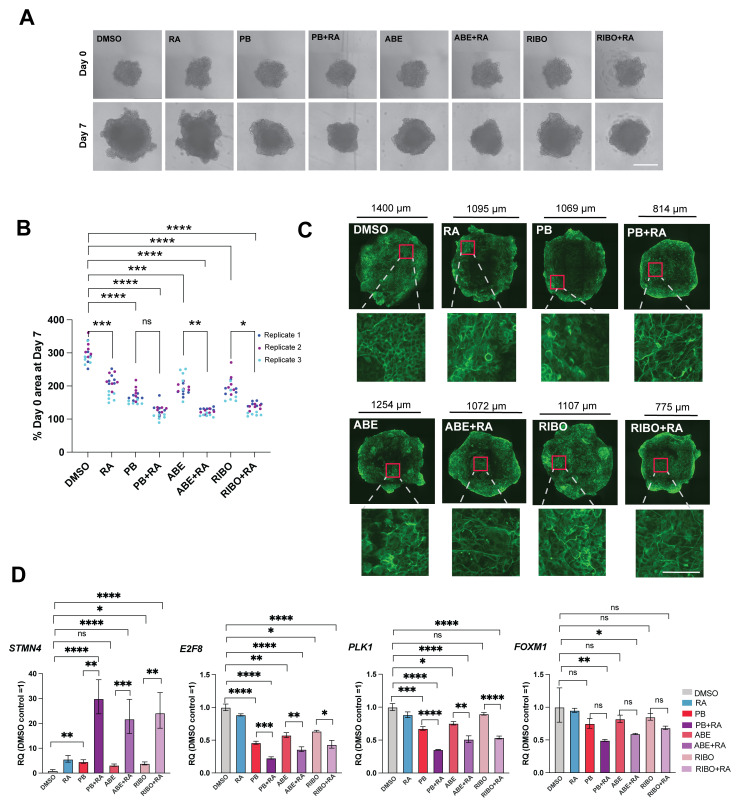
The CDK4/6 inhibitors palbociclib, abemaciclib and ribociclib all enhance retinoic acid-induced differentiation in 3D SK-N-BE(2)C spheroids. ( **A**) Representative phase-contrast images of SK-N-BE(2)C spheroids at day 0 and day 7 of treatment with palbociclib (PB, 1 μM), abemaciclib (ABE, 0.2 μM), ribociclib (RIBO, 2 μM), retinoic acid (RA, 10 μM) or a combination of PB/ABE/RIBO + RA, or DMSO vehicle control, at the same concentrations used throughout the manuscript. Scale bar: 500 μm. ( **B**) Percentage SK-N-BE(2)C spheroid area at day 7 of treatment compared with day 0. n = 3 biological replicates, with n = 6 spheroids per replicate, each represented by a single data point. ^*^p ≤ 0.05; ^**^p ≤ 0.01, ^***^p ≤ 0.001; and ^****^p ≤ 0.0001, one-way ANOVA with Tukey’s multiple comparison test. Selected comparisons shown for ease of visualisation. ( **C**) Immunofluorescence images of tumour spheroids stained for neuronal marker βIII-tubulin (TUBB3, green) at day 7 of treatment. Scale shown for each individual image. Higher magnification images shown with scale bar: 100 μm. Representative of n=3 biological replicates. ( **D**) qRT-PCR analysis of *STMN4, E2F8, PLK1* and *FOXM1* expression levels in SK-N-BE(2)C spheroids treated with palbociclib (PB), abemaciclib (ABE), ribociclib (RIBO), retinoic acid (RA) or a combination of PB/ABE/RIBO + RA, or DMSO vehicle control for 7 days (∼30 spheroids pooled per replicate, n = 3 biological replicates). Mean ± 95% confidence interval (CI). ^*^p ≤ 0.05; ^**^p ≤ 0.01, ^***^p ≤ 0.001; and ^****^p ≤ 0.0001, one-way ANOVA with Tukey’s multiple comparison test. Selected comparisons shown for ease of visualisation.

Finally, we wanted to assess the effect of CDK4/6 inhibition on multicellular 3D tumour spheroids. We found a decrease in spheroid growth upon treatment with each CDK4/6 inhibitor compared to DMSO, enhanced by addition of retinoic acid ( Figure 3 A,B). Immunostaining of spheroids for the neuronal marker TUBB3 revealed morphological changes to cells and an increase in neurite extension with CDK4/6 inhibitor and CDK4/6i+RA treatment ( Figure 3C), compared to DMSO-treated controls. Finally, qRT-PCR analysis revealed a consistent pattern of a greater increase in *STMN4* expression, and a greater decrease in *E2F8, PLK1* and *FOXM1* expression, upon treatment of spheroids with each CDK4/6 inhibitor plus RA, compared to treatment with each CDK4/6 inhibitor alone ( Figure 3D). In summary, we find that CDK4/6 inhibitors display a class effect in reducing proliferation and inducing neuronal differentiation together with retinoic acid, both in two-dimensional and three-dimensional *in vitro* neuroblastoma cell cultures. 

## Discussion

We conclude that treatment of the neuroblastoma cell line SK-N-BE(2)C with any CDK4/6 inhibitor (palbociclib, abemaciclib or ribociclib) at IC50 concentrations results in RB hypo-phosphorylation and both reduces proliferation and induces differentiation, without extensive cell death. Our results demonstrate that combining any of these CDK4/6 inhibitors with retinoic acid (RA) further inhibits cell cycling and enhances differentiation of SK-N-BE(2)C cells in 2D and 3D, compared to treatment with each CDK4/6 inhibitor alone. We therefore conclude this is a class effect; all CDK4/6 inhibitors have a combinatory effect with retinoic acid in inducing differentiation of neuroblastoma cells.

It would be interesting to determine the functionality of the cells following CDK4/6 inhibitor + RA treatment and we invite this for further research. Previously, we observed PB+RA treatment induced ultrastructural features reminiscent of mature neuronal cells, in particular dense-core granules ^2^. Additional analyses could include assessment of electrophysiological functions and cell-to-cell signal transduction. Importantly, the use of neuronal cell culture media or more complex in vivo systems should be considered, as the microenvironment is likely to influence the ability of differentiated cells to acquire electrophysiological neuronal functions. Such assessments could be useful for fields such as Parkinson’s disease pathobiology, where differentiation of SH-SY5Y cells with RA alone is used as a model system ^18^. While we compared each CDK4/6 inhibitor to the DMSO vehicle control to determine if they exhibit a class-effect, comparing the efficacy of each CDK4/6 inhibitor to one another is also an interesting future research question.

CDK inhibitors have been extensively investigated for their ability to arrest proliferation in cancers where cell cycle components are dysregulated. Previously, we hypothesised that by lengthening the G1 phase of the cell cycle, palbociclib may provide a ‘phenotype switch’ trigger by increasing the available time for cell response to differentiation cues ^2, 19, 20^, based on the cell-cycle length model ^19^. Indeed, Calegari *et al.* ^19, 20^ provide evidence that G1 lengthening casually induces neuroepithelial differentiation of proliferative neural progenitors. Reduction of serum in cell culture media induces G1 arrest and is often used in combination with retinoic acid to induce neuroblastoma cell differentiation *in vitro* ^21^; we therefore surmised that palbociclib was providing a similar, albeit more potent, effect in triggering differentiation. Here, we find that three independent CDK4/6 inhibitors all induce neuroblastoma differentiation, confirming the hypothesis that G1 lengthening is a key mechanistic trigger for this process. This correlation between the cell cycle, in particular G1, and spatial and temporal regulation of cell fate determination has been reviewed across several model systems and stem cell lineages ^22– 24^.

Importantly, all three inhibitors show a combinatorial effect in enhancing differentiation when combined with retinoic acid, already used in maintenance therapy to treat minimal residual disease in high-risk neuroblastoma cases. On potentially developing CDK inhibitor-induced differentiation as a therapy in the clinic, it will therefore be important to consider CDK4/6 inhibitors as a class, balancing their differentiation activity with factors such as availability, cost, pharmacodynamics and toxicity profiles.

## Ethics and consent statement

Ethical approval and consent were not required.

## Data Availability

Zenodo: CDK4/6 inhibitors display a class effect in inducing differentiation of neuroblastoma cells. https://doi.org/10.5281/zenodo.13850646 ^25^. This project contains the following underlying data: Data for Figure 1A–D, Figure 2A–F and Figure 3A–D. Figure1A: Western Blot for RB and pRB with TBP loading control. Figure1B and Figure2A: Representative phase-contrast images. Figure1C and Figure2B: Representative crystal violet staining images. Figure1D and Figure 2C: Raw representative immunocytochemistry images, TUBB3 and DAPI staining. Figure2D: GraphPad Prism file of raw % EdU counts and statistical analysis. Figure2E: GraphPad Prism file of normalised confluency data from Incucyte® live-imaging and statistical analysis. Figure2F and Figure3D: GraphPad Prism file of raw qRT-PCR ddCt values, Mean ± 95% CI values and statistical analysis. Figure3A: Representative phase-contrast images of spheroids at Day 0 and Day 7. Figure3B: GraphPad Prism file of normalised spheroid areas and statistical analysis. Figure3C: Raw representative immunocytochemistry images of spheroids, TUBB3 staining. Zenodo: CDK4/6 inhibitors display a class effect in inducing differentiation of neuroblastoma cells. https://doi.org/10.5281/zenodo.13850646 ^25^. This project contains the following extended data: Figure S1: related to Figure 1 (IC50 analysis for ABE and RIBO, phase-contrast and crystal violet images of SH-EP treated with PB, ABE, RIBO or DMSO vehicle control for 5 days), legends for Movie S1 and S2, and Table S1: qRT-PCR primer sequences. FigureS1A: GraphPad Prism file of IC50 value calculation based on % confluency. FigureS1B: Representative phase-contrast images. FigureS1C: Representative crystal violet staining images. Movie S1: Live imaging of SK-N-BE(2)C cells treated with PB, ABE, RIBO or DMSO vehicle control for 5 days. Related to Figure 1. Note: Figure 1 data forms part of dataset shown in Figure 2. Movie S2: Live imaging of SK-N-BE(2)C cells treated with PB, ABE, RIBO, RA or a combination of PB/ABE/RIBO + RA, or DMSO vehicle control for 5 days, related to Figure 2. Data are available under the terms of the Creative Commons Attribution 4.0 International license (CC-BY 4.0).

## References

[1] Brodeur GM : Spontaneous regression of neuroblastoma. *Cell Tissue Res.* 2018;372(2):277–286. 10.1007/s00441-017-2761-2 29305654 PMC5920563

[2] Ferguson KM GillenSL, Chaytor L : Palbociclib releases the latent differentiation capacity of neuroblastoma cells. *Dev Cell.* 2023;58(19):1967–1982. e8. 10.1016/j.devcel.2023.08.028 37734383 PMC7618569

[3] Klein ME Kovatcheva M Davis LE : CDK4/6 inhibitors: the mechanism of action may not be as simple as once thought. *Cancer Cell.* 2018;34(1):9–20. 10.1016/j.ccell.2018.03.023 29731395 PMC6039233

[4] Toogood PL Harvey PJ Repine JT : Discovery of a potent and selective inhibitor of cyclin-dependent kinase 4/6. *J Med Chem.* 2005;48(7):2388–2406. 10.1021/jm049354h 15801831

[5] Fry DW Harvey PJ Keller PR : Specific inhibition of cyclin-dependent kinase 4/6 by PD 0332991 and associated antitumor activity in human tumor xenografts. *Mol Cancer Ther.* 2004;3(11):1427–1438. 10.1158/1535-7163.1427.3.11 15542782

[6] Saab R Bills JL Miceli AP : Pharmacologic inhibition of Cyclin-dependent kinase 4/6 activity arrests proliferation in myoblasts and rhabdomyosarcoma-derived cells. *Mol Cancer Ther.* 2006;5(5):1299–1308. 10.1158/1535-7163.MCT-05-0383 16731763

[7] Marzec M Kasprzycka M Lai R : Mantle cell lymphoma cells express predominantly cyclin D1a isoform and are highly sensitive to selective inhibition of CDK4 kinase activity. *Blood.* 2006;108(5):1744–1750. 10.1182/blood-2006-04-016634 16690963 PMC1895502

[8] Rugo HS Finn RS Diéras V : Palbociclib plus letrozole as first-line therapy in estrogen receptor-positive/human epidermal growth factor receptor 2-negative advanced breast cancer with extended follow-up. *Breast Cancer Res Treat.* 2019;174(3):719–729. 10.1007/s10549-018-05125-4 30632023 PMC6438948

[9] Kim S Tiedt R Loo A : The potent and selective cyclin-dependent kinases 4 and 6 inhibitor ribociclib (LEE011) is a versatile combination partner in preclinical cancer models. *Oncotarget.* 2018;9(81):35226–35240. 10.18632/oncotarget.26215 30443290 PMC6219668

[10] Asghar U Witkiewicz AK Turner NC : The history and future of targeting cyclin-dependent kinases in cancer therapy. *Nat Rev Drug Discov.* 2015;14(2):130–146. 10.1038/nrd4504 25633797 PMC4480421

[11] Hortobagyi GN Stemmer SM Burris HA : Ribociclib as first-line therapy for HR-positive, advanced breast cancer. *N Engl J Med.* 2016;375(18):1738–1748. 10.1056/NEJMoa1609709 27717303

[12] Vinci M Gowan S Boxall F : Advances in establishment and analysis of three-dimensional tumor spheroid-based functional assays for target validation and drug evaluation. *BMC Biol.* 2012;10: 29. 10.1186/1741-7007-10-29 22439642 PMC3349530

[13] Connell-Crowley L Harper JW Goodrich DW : Cyclin Dl/Cdk4 regulates retinoblastoma protein- mediated cell cycle arrest by site-specific phosphorylation. *Mol Biol Cell.* 1997;8(2):287–301. 10.1091/mbc.8.2.287 9190208 PMC276080

[14] Rader J Russell MR Hart LS : Dual CDK4/CDK6 inhibition induces cell-cycle arrest and senescence in neuroblastoma. *Clin Cancer Res.* 2013;19(22):6173–6182. 10.1158/1078-0432.CCR-13-1675 24045179 PMC3844928

[15] Zimmerman MW Durbin AD He S : Retinoic acid rewires the adrenergic core regulatory circuitry of childhood neuroblastoma. *Sci Adv.* 2021;7(43): eabe0834. 10.1126/sciadv.abe0834 34669465 PMC8528416

[16] Pajtler KW Sadowski N Ackermann S : The GSK461364 PLK1 inhibitor exhibits strong antitumoral activity in preclinical neuroblastoma models. *Oncotarget.* 2017;8(4):6730–6741. 10.18632/oncotarget.14268 28036269 PMC5351666

[17] Wang Z Park HJ Carr JR : *FoxM1* in Tumorigenicity of the neuroblastoma cells and renewal of the neural progenitors. *Cancer Res.* 2011;71(12):4292–4302. 10.1158/0008-5472.CAN-10-4087 21507930 PMC3771352

[18] Xicoy H Wieringa B Martens GJM : The SH-SY5Y cell line in Parkinson's Disease research: a systematic review. *Mol Neurodegener.* 2017;12(1):10. 10.1186/s13024-017-0149-0 28118852 PMC5259880

[19] Calegari F Huttner WB : An inhibition of cyclin-dependent kinases that lengthens, but does not arrest, neuroepithelial cell cycle induces premature neurogenesis. *J Cell Sci.* 2003;116(Pt 24):4947–4955. 10.1242/jcs.00825 14625388

[20] Calegari F : Selective lengthening of the cell cycle in the neurogenic subpopulation of neural progenitor cells during mouse brain development. *J Neurosci.* 2005;25(28):6533–6538. 10.1523/JNEUROSCI.0778-05.2005 16014714 PMC6725437

[21] Rawson C Loo D Helmrich A : Serum inhibition of proliferation of Serum-Free Mouse Embryo cells. *Exp Cell Res.* 1991;192(1):271–277. 10.1016/0014-4827(91)90186-x 1898591

[22] Hardwick LJA Ali FR Azzarelli R : Cell cycle regulation of proliferation versus differentiation in the central nervous system. *Cell Tissue Res.* 2015;359(1):187–200. 10.1007/s00441-014-1895-8 24859217 PMC4284380

[23] Hindley C Philpott A : Co-ordination of cell cycle and differentiation in the developing nervous system. *Biochem J.* 2012;444(3):375–82. 10.1042/BJ20112040 22642576 PMC3365434

[24] Lange C Calegari F : Cdks and cyclins link G _1_ length and differentiation of embryonic, neural and hematopoietic stem cells. *Cell Cycle.* 2010;9(10):1893–900. 10.4161/cc.9.10.11598 20436288

[25] Ferguson KM Abou Grealy FMY Philpott A : CDK4/6 inhibitors display a class effect in inducing differentiation of neuroblastoma cells. [Data set]. Zenodo. 2024. 10.5281/zenodo.13850646 PMC12553970 41146951

