# Supplementary material for: CDK4/6 inhibitors display a class effect in inducing differentiation of neuroblastoma cells
Source: Wellcome Open Res. 2025 Sep 3;9:667. Originally published 2024 Nov 8. [Version 2] doi: 10.12688/wellcomeopenres.23190.2 (PMC12553970; doi:10.12688/wellcomeopenres.23190.2)
Supplement: Peer review history

# Reviewer report: Reviewer response for version 2

**DOI:** 10.21956/wellcomeopenres.27410.r133771

**Published:** 2025-10-25

**Authors:** Marie Arsenian-Henriksson, Mohammad Alzrigat

**Affiliations:** 1 Karolinska Institutet, Stockholm, Sweden; 2 MTC, Karolinska Institutet Institutionen for neurovetenskap, Stockholm, Stockholm County, Sweden

In this manuscript Ferguson KM, et al. investigated the impact of different CDK4/6 inhibitors on the differentiation of neuroblastoma cells. This study is a follow up of their previously elegant study: ‘’Ferguson KM, et al. Palbociclib releases the latent differentiation capacity of neuroblastoma cells. Dev Cell. 2023 Oct 9;58(19):1967-1982.e8. doi: 10.1016/j.devcel.2023.08.028. Epub 2023 Sep 20’’ in which they described at the molecular and cellular level how the CDK4/6 inhibitor Palbociclib induces neuronal differentiation in adrenergic neuroblastoma cell lines. Thus, the novelty of this study is somewhat diminished by their previous findings – both the previous article and the current manuscript show that CDK4/6 inhibition induces a reduction in the proliferation and enhance neuronal differentiation of neuroblastoma cells alone or in combination with retinoic acid.

 However, since this study  as well as the previous work demonstrate for the first time that CDK4/6 inhibition induces neuronal differentiation, this manuscript is suitable for indexing after addressing the following concerns:

**Major concerns:**

1. Only one cell line of each neuroblastoma subtype was used in this study. The concept of using several cell lines has been shown previously in the Developmental Cell Paper. Still, please include more cell lines in the current study.
2. It is of great importance to the neuroblastoma scientific community to show CDK4 and CDK6 as well as their cell cycle regulated inhibitors at the protein levels in cell lines of MES and ADRN subtypes. Importantly, analysis of Cyclin D1 levels (control versus treated cells) would be of great addition to the results.
3. Considering the impact of cell cycle phases on biological responses to CDKi, the study would benefit from performing differentiation analysis following CDK4/6 inhibition on synchronized cells. This might shed new insights on the impact of cell cycle on response outcome to CDK4/6 inhibition as well as RA treatment.
4. Withdrawal of CDK4/6i, RA, or combination treatments after 5 or 7-days treatment is of great addition to this current manuscript as well as the previous study. It ids important for its translation into clinical practice to know whether neuronal differentiation is reversed upon stopping treatment. Will all cells differentiate? Will non-differentiated cells take over?

**Comments Related to Figure 1:**

 The authors suggest that the different CDK4/6 inhibitors demonstrated on target effect by reduction of p-RB and total-RB. The on-target effects should only be stated once there is reduction in p-RB without affecting total-RB protein levels. To this end, please analyze earlier time points i.e., 2, 6, 12, and 24 hours to check for on-target effects. Based on the presented data, the reduction in p-RB could be a consequence of reduced levels in total-RB. This also should be done on other ADRN neuroblastoma cell lines like in the previous study.

 It is important to state which phosphorylation site has been used to indicate CDK4/6 inhibition. This information is missing in the results, the materials and methods as well as in the discussion.

 Treatment of several MES cell lines with their IC50 of CDK4/6is followed by checking on-target effects on p-RB and total-RB is required to uncover if the lack of response in MES cell lines is due lack of effect of the CDKis.

 Have the authors treated ADNR and MES for longer time points – longer than 5-days to analyze whether the difference in neuronal differentiation capacity is maintained. Possibly the MES cell lines need longer treatment duration to show response to CDK4/6 inhibition.

**Comments Related to Figure 2:**

 The combination treatment and outcome on differentiation should also be performed on MES cell lines. Maybe under combinatorial settings MES cell lines might undergo neuronal differentiation.

 RA has the least impact on confluency despite the high 10 uM concentration used, which is translated by no impact of the expression of E2F8, PLK1 and FOXM1. Images show heterogeneity in BrdU staining. In addition, the authors reflected in prolongation of cell cycle to enhance differentiation. These issues could be addressed possibly by repeating these experiments on synchronized cells which might can help to understand the RA response as well as combinations in ADRN and MES cell lines.

 Have the authors analyzed STMN4, E2F8, PLK1 and FOXM1protein levels to make sure that regulation at mRNA levels is translated at the protein level?

**Comments Related to Figure 3.**

 Please add a figure showing the spheroid size/area relative to DMSO at day 0 and day 7. This way readers can appreciate the impact of single verss combination treatments on spheroid size/area.

 The black areas in the heart of some of some of the spherids – are they results of resolution, magnification of the image? Or are they areas that did not undergo neuronal differentiation? If so, is that due to the inefficacy of drug penetration or hypoxia, or cell death?

**Other comment related to all Figures:**

 Quantification of neurite number and length would improve the results and define the impact of each inhibitor alone or in combination on differentiation. RA should be used as positive reference.

Is the work clearly and accurately presented and does it cite the current literature?

Yes

If applicable, is the statistical analysis and its interpretation appropriate?

Yes

Are all the source data underlying the results available to ensure full reproducibility?

Yes

Is the study design appropriate and is the work technically sound?

Yes

Are the conclusions drawn adequately supported by the results?

Yes

Are sufficient details of methods and analysis provided to allow replication by others?

Yes

Reviewer Expertise:

Childhood cancer (neuroblastoma, medulloblastoma) and adult cancer (renal and cervical cancer). Our reserach focus on targeting MYC and metabolism as well as studies on neuronal differentiation (neuroblastoma and medulloblöastoma).

We confirm that we have read this submission and believe that we have an appropriate level of expertise to confirm that it is of an acceptable scientific standard, however we have significant reservations, as outlined above.

## References

[1] : Palbociclib releases the latent differentiation capacity of neuroblastoma cells. *Developmental Cell* .2023;58(19) : 10.1016/j.devcel.2023.08.028 1967-1982.e8 10.1016/j.devcel.2023.08.028 PMC761856937734383

# Reviewer report: Reviewer response for version 1

**DOI:** 10.21956/wellcomeopenres.25540.r110967

**Published:** 2024-11-29

**Authors:** Yang Li

**Affiliations:** 1 Sun Yat-Sen University, Guangdong, China

This study reveals that CDK4/6 inhibitors induce differentiation in NB cells and, when combined with RA, synergistically enhance differentiation. As CDK4/6 inhibitors are already clinically approved, these findings offer a potential novel therapeutic strategy for differentiation therapy during the maintenance phase in patients with high-risk neuroblastoma, highlighting important clinical implications.

 1.Western blot results show that abemaciclib, ribociclib, and palbociclib effectively inhibit CDK4 expression and RB phosphorylation. However, the mechanism underlying the reduction in total RB protein expression remains unclear. Additionally, the lack of quantitative WB band analysis limits the ability to directly compare the inhibitory effects of the three drugs.

 2.The absence of functional validation experiments prevents the determination of whether the induced differentiated cells exhibit genuine neuronal functions or if the differentiation state of NB cells is temporary.

 3.This study primarily focuses on cellular-level experiments and lacks supporting in vivo data.

 4.This study demonstrates that CDK4/6 inhibitors induce differentiation in NB cells; however, the underlying mechanisms have not been thoroughly investigated.

 5.The rationale with which the doses of the two drugs were chosen is not clear. A more in-depth study exploring a broad range of concentrations should be done. Furthermore, the assessment of a potential synergistic effect between RA and CDK4/6 inhibitors should be evaluated using appropriate synergy models (i.e. Chou-Talalay model or Bliss Excess).

Is the work clearly and accurately presented and does it cite the current literature?

Yes

If applicable, is the statistical analysis and its interpretation appropriate?

Yes

Are all the source data underlying the results available to ensure full reproducibility?

Yes

Is the study design appropriate and is the work technically sound?

Yes

Are the conclusions drawn adequately supported by the results?

Yes

Are sufficient details of methods and analysis provided to allow replication by others?

Yes

Reviewer Expertise:

Tumor chemotherapy and tumor immunity

I confirm that I have read this submission and believe that I have an appropriate level of expertise to confirm that it is of an acceptable scientific standard.

# Response

**Published:** 2025-08-23

**Authors:** Anna Philpott

**Affiliations:** Cambridge Stem Cell Institute, Cambridge, England, UK

We thank the reviewer for their helpful comments and respond to each of these below.  

- **Comment 1: **Western blot results show that abemaciclib, ribociclib, and palbociclib effectively inhibit CDK4 expression and RB phosphorylation. However, the mechanism underlying the reduction in total RB protein expression remains unclear. Additionally, the lack of quantitative WB band analysis limits the ability to directly compare the inhibitory effects of the three drugs.
- **Author response: **To confirm successful CDK4/6 inhibition, we analysed reduced phosphorylation of RB, a tumour suppressor that blocks the G1/S transition. The aim of this research article is to determine if the three CDK4/6 inhibitors, palbociclib, ribociclib and abemaciclib, display a class effect in inducing differentiation together with retinoic acid. For this reason, do not make direct comparisons between the CDK4/6 inhibitors, only comparison of each CDK4/6 inhibitor to its respective control, indeed observing that PB, RIBO or ABE treatment for 24h resulted in RB hypo-phosphorylation. This result is very clear visually and therefore we do not quantify the Western blot bands. It is indeed an interesting future research question to compare the efficacy of each CDK4/6 inhibitor to one another, beyond the aims of this manuscript. We have added this future research question to the Discussion (end of paragraph 2). Regarding the reduction in total RB, this observation has been previously reported in neuroblastoma cells upon CDK4/6 knock-down (PMID: 24045179, Figures 2 and 3); this was noted already of interest in the text and we invite others to investigate the molecular mechanisms for this decrease.

 

- **Comment 2: **The absence of functional validation experiments prevents the determination of whether the induced differentiated cells exhibit genuine neuronal functions or if the differentiation state of NB cells is temporary.
- **Author response: **We agree with the reviewer that it would be very interesting to investigate the functions of the differentiated cells, such as electrophysiological analyses. In our previous manuscript (PMID: 37734383) we performed extensive transcriptomic and epigenomic studies on PB+RA treated cells and “a mature differentiated state was observed both transcriptionally, by increased expression of neuronal differentiation features, and functionally by the increased presence of dense-core neurosecretory granules as visualized by TEM”. As the aim of our work is to investigate CDK4/6i+RA as a treatment in the paediatric cancer neuroblastoma, our priority, first and foremost, is to assess markers of differentiation and reduction in cell cycle, with the aim of halting tumour growth and enhance the current differentiation therapy of retinoic acid (RA). We invite further research into the functionality of the resulting neurons following CDK4/6i+RA treatment, for biological and clinical interest. It is possible that use of neuronal cell culture media and/or a more complex *in vivo* microenvironment would influence that ability of the differentiated cells to possess electrophysiological neuronal functions. This could be useful for fields such as Parkinson’s disease pathobiology where differentiated SH-SY5Y treated with retinoic acid alone are used as a model system (e.g. PMID: 28118852). We have now highlighted this potential future work in the Discussion text (paragraph 3). Regarding the stability of the differentiation state of the neuroblastoma cells, we began to further explore the differentiation potential of neuroblastoma cell lines *in vitro* in response to palbociclib and retinoic acid treatment in another manuscript ( https://wellcomeopenresearch.org/articles/9-671). It would indeed be very interesting to confirm if the differentiation state of the cells is terminally stable, and the treatment regime required to achieve this; such an experiment would be best performed in an *in vivo* system where the neuroblastoma cells are exposed to the tumour microenvironment and we will keep this in mind for future exploration leading to clinical translation.

 

- **Comment 3: **This study primarily focuses on cellular-level experiments and lacks supporting in vivo data.
- **Author response: **We agree it would be very interesting to test these inhibitors in vivo. Indeed, we are undertaking this as part of a separate study to provide supporting data for clinical application of CDK4/6 inhibitors+RA in treating neuroblastoma, but this work is beyond the scope of this paper.

 

- **Comment 4: **This study demonstrates that CDK4/6 inhibitors induce differentiation in NB cells; however, the underlying mechanisms have not been thoroughly investigated.
- **Author response: **In our previous manuscript published in Developmental Cell (PMID: 37734383), we performed extensive analyses to show that PB resets the global transcriptional and epigenetic landscapes of ADRNB-type neuroblastoma cells towards a differentiated state. As discussed in the manuscript, “lengthening the cell cycle may be the trigger for this phenotypic switch; for example, G1 lengthening is thought to increase the time available for cells to respond to differentiation cues”. As referenced, Calegari et al (PMID: 16014714 and PMID: 14625388) show that G1 lengthening casually induces neuroepithelial differentiation of proliferative neural progenitors. They propose a cell-cycle length model, where “a cell fate determinant may or may not cause a cell fate change, depending on whether the length of time this determinant can act is sufficient” and indeed show that neuroepithelial and radial glial cells in mouse embryos have a longer cell cycle than proliferating progenitors (PMID: 16014714). The effect of cell cycle on lineage commitment has been extensively investigated by Philpott lab and others (PMID: 24859217, PMID: 12593979, PMID: 24791612). We have now expanded on this point in the discussion to address the reviewers’ question (Discussion, paragraph 3).

 

- **Comment 5: **The rationale with which the doses of the two drugs were chosen is not clear. A more in-depth study exploring a broad range of concentrations should be done. Furthermore, the assessment of a potential synergistic effect between RA and CDK4/6 inhibitors should be evaluated using appropriate synergy models (i.e. Chou-Talalay model or Bliss Excess).
- **Author response: **As in our previous manuscript (PMID: 37734383), cells were treated 1 μM PB; this is a standard dosage used in cellular studies, and is similar to the IC50s that we established for all three lines (Figure S1A of PMID: 37734383). We therefore ascertained and used ribociclib and abemaciclib at their IC50 concentrations in SK-N-BE(2)C cells (2 μM and 0.2 μM, respectively). Cells were treated with all-trans retinoic acid (RA) at a concentration of 10 μM, commonly used in studies to differentiate SH-SY5Y neuroblastoma cells (e.g. PMID: 35205034).  Importantly, we confirmed that none of these dosages resulted in cell death. Thank you for the insightful comment on determining drug synergy; this is indeed important for clinical translation to reduce toxic effects and will be determined for *in vivo *assessment of CDK4/6i+RA.

  We thank the reviewer for their helpful comments which have guided submission of version 2 of this manuscript. As further experiments cannot be completed at this time as key staff have left the lab, we ask that the reviewer considers this in their responses.

# Reviewer report: Reviewer response for version 1

**DOI:** 10.21956/wellcomeopenres.25540.r110971

**Published:** 2024-11-15

**Authors:** Feng-Hou Gao

**Affiliations:** 1 Shanghai Jiao Tong University School of Medicine, Shanghai, China

This research focuses on cell differentiation therapy, aiming to redirect cancer cells back to the normal developmental pathway, thus providing new ideas for the treatment of neuroblastoma. In the field of neuroblastoma, previous studies have found that the CDK4/6 inhibitor palbociclib can induce a reduction in the proliferation and an enhancement in the neuronal differentiation of neuroblastoma cells, and the effect is even more significant when combined with retinoic acid. However, the roles of other CDK4/6 inhibitors in the differentiation of neuroblastoma cells remain unclear. This study was carried out under such a background and is of certain innovativeness and exploratory nature.

 1. In the Western blot results, only the protein bands were presented, but the quantitative data of phosphorylated RB and total RB protein levels were not clearly provided, making it impossible to accurately judge the differences in the degree of inhibition. For example, although the changing trends between the treatment groups and the control group could be observed, it was difficult to determine whether there were significant quantitative differences in inhibiting RB phosphorylation among abemaciclib, ribociclib and palbociclib, which was insufficient for an in-depth comparison of the effects of different inhibitors.

 2. Although the phase contrast microscope images (Figure 1B) could observe the changes in cell morphology, no quantitative description was made of these changes, such as the measurement and statistical analysis of cell size, neurite length and other characteristics. Merely through image observation, it was difficult to accurately compare the degree and characteristics of cell morphology changes, which might affect the accurate assessment of the differentiation-inducing effects of the inhibitors.

 3. The experiment only set DMSO solvent as the control, without setting a positive control (such as other drugs or treatment methods known to be able to effectively induce the differentiation of neuroblastoma cells). This made it difficult to directly compare when evaluating the differentiation-inducing effects of CDK4/6 inhibitors, and it was hard to determine the strength of their effects and their positions in similar studies, which was not conducive to a comprehensive evaluation of the reliability and innovativeness of the research results.

 4. In the EdU analysis (Figure 2D), although the analysis results of %EdU positive cells were provided, it was not clearly stated how EdU positive cells were defined, for example, whether a specific fluorescence intensity threshold was set. This made it difficult for other researchers to accurately repeat the experiment and evaluate the reliability of the data. In addition, only some comparisons between treatment groups were presented in the figure, without completely showing all possible pairwise comparison results, which might affect the comprehensive understanding of the differences between different treatment groups.

 5. In the immunofluorescence staining images (Figure 3C), although the expression of TUBB3 in tumor spheroid cells was shown, the resolution of some images was not high enough, making it difficult to clearly identify the cell details, such as the fine structures of neurites and the intercellular relationships. Moreover, there was no clear labeling of cell types or regions in the images, making it difficult for readers to accurately judge the specific cell locations and distribution of the enhanced TUBB3 expression, thus affecting the accurate assessment of the cell differentiation state.

 6. Although the effects of the inhibitors on tumor spheroid growth and cell differentiation were confirmed by multiple methods, further functional verification experiments were lacking. For example, the functions of the differentiated cells were not detected, such as the electrophysiological functions specific to neurons and cell-to-cell signal transduction. This made the assessment of cell differentiation only stay at the morphological and gene expression levels, and it was impossible to determine whether the differentiated cells had true neuronal functions, which limited the comprehensive understanding of the differentiation-inducing effects of the inhibitors and their evaluation in potential therapeutic applications.

 7. The experiment mainly focused on the research at the cell level, lacking in vivo experimental data. The in vivo environment is complex, and factors such as cell-cell interactions and immune responses may affect the effects of the inhibitors. Therefore, it is difficult to directly infer the effectiveness of the inhibitors in clinical treatment solely based on the in vitro experimental results.

 8. Although the aspects such as cell proliferation and differentiation were studied, the specific molecular mechanisms by which CDK4/6 inhibitors induce the differentiation of neuroblastoma cells were not thoroughly explored. For example, it remains unclear how the prolongation of the G1 phase triggers the molecular signaling pathways of differentiation, which limits the in-depth understanding and further optimization of this therapy.

Is the work clearly and accurately presented and does it cite the current literature?

Partly

If applicable, is the statistical analysis and its interpretation appropriate?

Yes

Are all the source data underlying the results available to ensure full reproducibility?

Yes

Is the study design appropriate and is the work technically sound?

Yes

Are the conclusions drawn adequately supported by the results?

Yes

Are sufficient details of methods and analysis provided to allow replication by others?

Yes

Reviewer Expertise:

Tumor microenvironment and tumor immunity。

I confirm that I have read this submission and believe that I have an appropriate level of expertise to confirm that it is of an acceptable scientific standard, however I have significant reservations, as outlined above.

# Response

**Published:** 2025-08-23

**Authors:** Anna Philpott

**Affiliations:** Cambridge Stem Cell Institute, Cambridge, England, UK

We thank the reviewer for their helpful comments and respond to each of these below.

**Comment 1**: In the Western blot results, only the protein bands were presented, but the quantitative data of phosphorylated RB and total RB protein levels were not clearly provided, making it impossible to accurately judge the differences in the degree of inhibition. For example, although the changing trends between the treatment groups and the control group could be observed, it was difficult to determine whether there were significant quantitative differences in inhibiting RB phosphorylation among abemaciclib, ribociclib and palbociclib, which was insufficient for an in-depth comparison of the effects of different inhibitors. **Author response**: To confirm successful CDK4/6 inhibition, we analysed reduced phosphorylation of RB, a tumour suppressor that blocks the G1/S transition. The aim of this research article is to determine if the three CDK4/6 inhibitors, palbociclib, ribociclib and abemaciclib, display a class effect in inducing differentiation together with retinoic acid. For this reason, we do not make direct comparisons between the CDK4/6 inhibitors, but compare each CDK4/6 inhibitor to the control. We observe that PB, RIBO or ABE treatment for 24h results in RB hypo-phosphorylation, compared to the DMSO-treated control. This result is very clear visually and therefore we do not quantify the Western blot bands. It is indeed an interesting future research question to compare the efficacy of each CDK4/6 inhibitor to one another, but is beyond the aims of this manuscript. We have added this future research question to the Discussion (end of paragraph 2).

**Comment 2:** Although the phase contrast microscope images (Figure 1B) could observe the changes in cell morphology, no quantitative description was made of these changes, such as the measurement and statistical analysis of cell size, neurite length and other characteristics. Merely through image observation, it was difficult to accurately compare the degree and characteristics of cell morphology changes, which might affect the accurate assessment of the differentiation-inducing effects of the inhibitors. **Author response**: In addition to the phase contrast images presented in Figure 1B, we also present crystal violet staining images (Figure 1C) and immunocytochemistry for the neuronal marker TUBB3 (Figure 1D) as supporting data. Figure 1C shows a striking difference in cell proliferation between the DMSO control and PB/ABE/RIBO-treated cells. Figure 1D provides complementary data to Figure 1B, showing a striking upregulation in TUBB3 expression upon PB/ABE/RIBO treatment, with significant changes in cell morphology, notably neurite extensions. We have also provided live imaging of SK-N-BE(2)C cells undergoing these drug treatments in the Extended Data Movie S1; neurite formation can be observed in real-time following drug treatment (with no neurite extension in the DMSO control), supporting the differentiation-inducing effects of the inhibitors.

**Comment 3: **The experiment only set DMSO solvent as the control, without setting a positive control (such as other drugs or treatment methods known to be able to effectively induce the differentiation of neuroblastoma cells). This made it difficult to directly compare when evaluating the differentiation-inducing effects of CDK4/6 inhibitors, and it was hard to determine the strength of their effects and their positions in similar studies, which was not conducive to a comprehensive evaluation of the reliability and innovativeness of the research results. **Author response:** Thank you for raising this point for discussion. Retinoic acid is used as a differentiation-inducing agent in neuroblastoma cells, and would therefore be considered a positive control. The effect of RA treatment alone can be seen in Figure 2 and Figure 3 of this manuscript, and can be considered a positive control for differentiation. However, as can be seen by our results here and in our previous publication (PMID: 37734383), the differentiation-inducing effects of retinoic acid are substantially enhanced by CDK4/6 inhibition. We have now clarified this in the text (Results section - “The CDK4/6 inhibitors palbociclib, abemaciclib and ribociclib all enhance retinoic acid-induced differentiation in adherent SK-N-BE(2)C cells”).

**Comment 4: **In the EdU analysis (Figure 2D), although the analysis results of %EdU positive cells were provided, it was not clearly stated how EdU positive cells were defined, for example, whether a specific fluorescence intensity threshold was set. This made it difficult for other researchers to accurately repeat the experiment and evaluate the reliability of the data. In addition, only some comparisons between treatment groups were presented in the figure, without completely showing all possible pairwise comparison results, which might affect the comprehensive understanding of the differences between different treatment groups. **Author response: **For the EdU analysis, FIJI image thresholding and particle analysis functions were used to quantify the % of EdU positive cells, as stated in the methods section using standard analysis methods. As standard for fluorescence imaging analysis, identical settings were used throughout the experiment for all treatment groups and replicates. This has now been further clarified in the Methods section. To aid readability and visualisation, only pairwise comparisons from which we draw conclusions are shown in each plot as stated in the figure legend (‘Selected comparisons shown for ease of visualisation’). However, for those interested in other condition comparisons, all comparisons for the one-way ANOVA with Tukey’s multiple comparison test can be viewed in the Underlying Data ‘Figure2D: GraphPad Prism file of raw % EdU counts and statistical analysis’ using the freely available GraphPad Prism Viewer.

**Comment 5: **In the immunofluorescence staining images (Figure 3C), although the expression of TUBB3 in tumor spheroid cells was shown, the resolution of some images was not high enough, making it difficult to clearly identify the cell details, such as the fine structures of neurites and the intercellular relationships. Moreover, there was no clear labeling of cell types or regions in the images, making it difficult for readers to accurately judge the specific cell locations and distribution of the enhanced TUBB3 expression, thus affecting the accurate assessment of the cell differentiation state. **Author response: **The reader can find original high resolution images of the whole spheroids and magnified regions in Underlying data ‘Figure3C: Raw representative immunocytochemistry images of spheroids, TUBB3 staining’. In this manuscript we have not determined intercellular relationships or cell locations, as the immunostaining was performed to visually demonstrate morphological changes to cells and an increase in neurite extension with CDK4/6 inhibitor and CDK4/6i+RA treatment. To provide quantitative data, we have performed qRT-PCR analyses of the neuronal differentiation marker STMN4, which shows a striking upregulation in expression in spheroids with CDK4/6i+RA treatment compared to the DMSO control.

**Comment 6: **Although the effects of the inhibitors on tumor spheroid growth and cell differentiation were confirmed by multiple methods, further functional verification experiments were lacking. For example, the functions of the differentiated cells were not detected, such as the electrophysiological functions specific to neurons and cell-to-cell signal transduction. This made the assessment of cell differentiation only stay at the morphological and gene expression levels, and it was impossible to determine whether the differentiated cells had true neuronal functions, which limited the comprehensive understanding of the differentiation-inducing effects of the inhibitors and their evaluation in potential therapeutic applications. **Author response: **We agree with the reviewer that it would be very interesting to investigate the functions of the differentiated cells, such as electrophysiological analyses. In our previous manuscript (PMID: 37734383) we performed extensive transcriptomic and epigenomic studies on PB+RA treated cells and “a mature differentiated state was observed both transcriptionally, by increased expression of neuronal differentiation features, and functionally by the increased presence of dense-core neurosecretory granules as visualized by TEM”. As the aim of our work is to investigate CDK4/6i+RA as a treatment in the paediatric cancer neuroblastoma, our priority, first and foremost, is to assess markers of differentiation and reduction in cell cycle, with the aim of halting tumour growth and enhance the current differentiation therapy of retinoic acid (RA). We invite further research into the functionality of the resulting neurons following CDK4/6i+RA treatment, for biological and clinical interest. It is possible that use of neuronal cell culture media and/or a more complex *in vivo* microenvironment would influence that ability of the differentiated cells to possess electrophysiological neuronal functions. This could be useful for fields such as Parkinson’s disease pathobiology where differentiated SH-SY5Y treated with retinoic acid alone are used as a model system (e.g. PMID: 28118852). We have now highlighted this potential future work in the Discussion text (paragraph 3).

**Comment 7: **The experiment mainly focused on the research at the cell level, lacking in vivo experimental data. The in vivo environment is complex, and factors such as cell-cell interactions and immune responses may affect the effects of the inhibitors. Therefore, it is difficult to directly infer the effectiveness of the inhibitors in clinical treatment solely based on the in vitro experimental results. **Author response: **We agree it would be very interesting to test these inhibitors in vivo. Indeed, we are undertaking this as part of a separate study to provide supporting data for clinical application of CDK4/6 inhibitors+RA in treating neuroblastoma, but this work is beyond the scope of this study.

**Comment 8: **Although the aspects such as cell proliferation and differentiation were studied, the specific molecular mechanisms by which CDK4/6 inhibitors induce the differentiation of neuroblastoma cells were not thoroughly explored. For example, it remains unclear how the prolongation of the G1 phase triggers the molecular signaling pathways of differentiation, which limits the in-depth understanding and further optimization of this therapy. **Author response: **Thank you for raising this for discussion. As discussed in the manuscript, “lengthening the cell cycle may be the trigger for this phenotypic switch; for example, G1 lengthening is thought to increase the time available for cells to respond to differentiation cues”. As referenced, Calegari et al (PMID: 16014714 and PMID: 14625388) show that G1 lengthening casually induces neuroepithelial differentiation of proliferative neural progenitors. They propose a cell-cycle length model, where “a cell fate determinant may or may not cause a cell fate change, depending on whether the length of time this determinant can act is sufficient” and indeed show that neuroepithelial and radial glial cells in mouse embryos have a longer cell cycle than proliferating progenitors (PMID: 16014714). The effect of cell cycle on lineage commitment has been extensively investigated by Philpott lab and others (PMID: 24859217, PMID: 12593979, PMID: 24791612), and further understanding of the link between G1 lengthening and differentiation is beyond the aims of this study. We have now expanded on this point in the discussion to address the reviewers’ question (Discussion, paragraph 3).

 We thank the reviewer for their helpful comments which have guided submission of version 2 of this manuscript. As further experiments cannot be completed at this time as key staff have left the lab, we ask that the reviewer considers this in their responses.
